# Effect of Fiber Loading on Green Composites of Recycled HDPE Reinforced with Banana Short Fiber: Physical, Mechanical and Morphological Properties

**DOI:** 10.3390/polym16233299

**Published:** 2024-11-26

**Authors:** Andres Felipe Rubiano-Navarrete, Pedro Rodríguez Sandoval, Yolanda Torres Pérez, Edwin Yesid Gómez-Pachón

**Affiliations:** 1 Grupo de Investigación en Diseño, Innovación y Asistencia Técnica para Materiales Avanzados-DITMAV, Maestría en Metalurgia y Ciencia de los Materiales, Universidad Pedagógica y Tecnológica de Colombia—UPTC, Tunja 150003, Colombia; 2 Grupo de Investigación de Materiales y Ensayos-GIMES, SENA-Centro de Materiales y Ensayos, Escuela de Posgrado en Ingeniería, Universidad Pedagógica y Tecnológica de Colombia—UPTC, Tunja 150003, Colombia; pedro.rodriguez01@uptc.edu.co; 3 Grupo de Investigación en Energía y Nuevas Tecnologías—GENTE, Escuela Ingeniería Electromecánica, Facultad Duitama, Universidad Pedagógica y Tecnológica de Colombia—UPTC, Duitama 150461, Colombia; yolanda.torres01@uptc.edu.co; 4 Grupo de Investigación en Diseño, Innovación y Asistencia Técnica para Materiales Avanzados-DITMAV, Escuela de Diseño Industrial, Universidad Pedagógica y Tecnológica de Colombia—UPTC, Duitama 150461, Colombia; edwin.gomez02@uptc.edu.co

**Keywords:** composite materials, materials science, mechanical strength, natural fibers, recycled materials

## Abstract

Currently, research on composite materials derived from natural fibers and agro-industrial waste has generated industrial proposals for producing useful materials with sufficient mechanical strength for applications involving the reuse of waste for secondary purposes. The objective of this study was to determine the influence of fiber content on the final tensile strength of the composite material, serving as a reference for the manufacture of plates. To achieve this, high-density polyethylene (HDPE) composites reinforced with short banana fibers were prepared using a blade mill and hot compression molding techniques. Two levels of short banana fiber content—10% and 20% by weight—were used, along with two types of HDPE: virgin and recycled. We evaluated the effect of adding short banana fibers on the mechanical properties of the composite, specifically tensile strength, according to the ASTM D638 standard for polymeric materials. These results were correlated with the structural properties obtained through morphological, chemical, and thermal characterization of the developed materials. The mechanical evaluation results showed that the tensile strength and elastic modulus depend on the short banana fiber content and the type of high-density polyethylene. Thermogravimetric analysis revealed that the composites decompose faster than the pure polymers (virgin and recycled HDPE). Based on these findings, the composite material prepared under optimal conditions is recommended for use in walls or construction boards where high tensile strength is not critical, due to the decreased mechanical properties resulting from the incorporation of agro-industrial waste.

## 1. Introduction

One of the priorities in moving towards a circular economy is to increase the efficient use of residual resources. This approach can reduce environmental pressure while enhancing competitiveness and innovation in the new economy, as well as promoting growth in productivity across various industrial and agro-industrial sectors [1]. The utilization of agro-industrial residues as resources aligns with the objectives of sustainable consumption and production.

The global dynamics of plastic usage are exemplified by the fact that one million plastic bottles are purchased every minute, and 500 billion plastic bags are used annually, with a staggering eight million tons of plastic waste ending up in the oceans each year [2,3,4,5]. According to a study by the Organization for Economic Cooperation and Development (OECD), 22% of plastic waste is improperly discarded worldwide, and only 9% is successfully recycled. Additionally, the annual per capita generation of plastic waste varies significantly: from 221 kg in the United States to 114 kg in OECD European countries and down to an average of just 69 kg in countries like Japan and South Korea [6,7].

Composite materials reinforced with fibers of natural origin or “NFRCs” (Natural Fiber Reinforced Composites) present different advantages from an economic and environmental perspective. This is due to the origin of the fibers, their abundance and the fact that they can be recycled, not to mention that some fibers are considered organic waste [8,9]. These factors can lead to a relatively low cost compared to fibers of synthetic origin, which are the most used in the automotive industry as reinforcing materials [10,11]. They are also employed in the manufacture of doors, seats, bodies, trunk, and bumpers, among others [12,13,14]. Therefore, it is essential to understand the mechanical efficiency of polymeric compositions.

Several studies have examined the mechanical properties of manufactured NFRCs, determining that the tensile strength of chemically treated banana fiber composites is higher than that of untreated fibers [12,15,16]. Research by Rahul Shrivastava indicated that alkali-treated short fibers exhibit higher tensile and flexural strength than long fibers in Coir/G Lass composites [17]. Hitoshi Takagi noted that natural fibers with the highest cellulose content and smallest microfibril angle are responsible for improving tensile properties [18]. The chemical composition of the fibers varies according to factors such as location, age, type, soil, rainfall, and cultivation methods. Generally, plant fibers consist of approximately 6–7% lignin, 10% pectin, 12% hemicellulose, and 71% cellulose, with the proportion of fibers varying from 10% to 40% [19]. Gupta M. indicated that natural plant fibers contain a high percentage of cellulose, which is mainly responsible for moisture absorption. Some cellulose microfibers are spirally wound and joined through an amorphous lignin matrix, playing a role in resistance to biological attack and providing strength [20]. Palanikumar K. and Ashok Kumar M. determined that alkaline treatment of vegetable fibers is decisive in increasing the tensile strength of polymeric compounds [21,22]. Previous research by Jaballi S., Miraoui I., and Hassis H. defined different weight percentages of composite materials reinforced with fully biodegradable polyvinyl alcohol, demonstrating improvements in their mechanical properties and suggesting suitability for applications in agriculture and packaging [23].

Banana fiber, a cellulose fiber produced from the pseudostem of the banana plant, boasts high specific strength qualities comparable to those of fiberglass [24], making it equivalent to conventional materials. The demand for natural fibers as renewable resources to strengthen polymeric composites is increasing. Annually, there is a 20% to 25% increase in automotive applications and more than a 55% increase in construction, with natural fibers also used as reinforcing elements in polymer compounds for tiles, furniture, and marine applications [24].

The technique used to create banana fiber composite material with specific mechanical characteristics is essential in determining its applications, as polymer composites are widely used in the automotive and construction industries, as well as in various climatic environments [25,26]. The choice of natural fibers not only aims to improve material properties but also reflects a commitment to reducing environmental impact. The use of banana fibers, an agricultural by-product, contributes to the reduction of agro-industrial waste, thus promoting a circular economy model. Moreover, when these fibers are added to a polymeric material, it reduces the amount of polyethylene in the final material, decreasing from 100% to 90% or 80% high-density polyethylene (HDPE).

From an energy perspective, incorporating natural fibers into HDPE allows significant optimization of energy consumption during the manufacturing process. As materials with low energy costs in production and processing, banana fibers contribute to reducing the carbon footprint associated with the final material. This approach not only enhances the sustainability of the material but also allows it to remain competitive in terms of cost and efficiency, making this composite a viable and environmentally responsible option.

Therefore, the present study focuses on evaluating the tensile strength of composite laminates produced from high-density polyethylene reinforced with different loads of short banana fibers. A morphological, thermal, and chemical characterization of the samples is carried out, continuing the research already published by Rubiano, Fabian, Pérez y Gómez [27,28].

## 2. Materials and Methods

In this study, experiments were conducted to develop and obtain materials for tensile testing, focusing on a polymeric matrix composite material reinforced with 10% and 20% short banana fibers. The composite materials were prepared using the following parameters and methodologies.

### 2.1. Preparation of Raw Material

Organic residues of banana bagasse from the *Musa paradisiaca* variety were obtained from the municipality of Moniquirá, Boyacá, Colombia, which has an average temperature of 19 °C and an altitude of 1700 m above sea level [29]. Short banana fibers were extracted from the pseudostem of the banana plant using a Trapp model JTRF70 grinder. The fibers were then washed to eliminate impurities and treated with a 5% NaOH alkaline solution for 1 h to remove lignin, following the methodology used by Praveen Nagarajan Durai [24]. After the alkaline treatment, the fibers were rinsed with distilled water and dried in a BIOBASE forced-air drying oven at 70 °C for 8 h to eliminate all traces of humidity.

Subsequently, the fibers were ground using a Gardom brand stainless-steel mill to reduce their size to below 5 mm in length, which was confirmed through sieving with a 5 mm sieve. The resulting fibers ranged between 0.2 and 5 mm in length. Recycled high-density polyethylene (HDPEr) pellets were procured from a local recycling plant, originating primarily from dairy waste.

The composite materials were fabricated by mixing the alkali-treated banana fibers and HDPEr using an EQUIPOL series 14,198 extruder (ref. PTL-30-30) at a mixing speed of 55 RPM and temperatures of 160 °C, 175 °C, 185 °C, and 210 °C to obtain a homogeneous mixture. These processing parameters were derived from theoretical and practical parameterization of the extruder. Three types of filament-shaped composite materials were produced: HDPEr + 10% BF (recycled HDPE reinforced with 10% banana fiber), HDPEr + 20% BF (recycled HDPE reinforced with 20% banana fiber), and HDPEv + 10% BF (virgin HDPE reinforced with 10% banana fiber). The fibers were randomly distributed within the polymeric matrix. These reinforcement percentages were based on established literature where high tensile strength was found, and the extrusion process was optimized to acquire the composite material [30].

The filaments were then pelletized using an EQUIPOL series 14,198 pelletizer (ref. PTL-30-30) to reduce the material size to a length of 5 mm. These pellets were spread into a metal mold with dimensions of 18 cm × 14 cm. The composite panels were fabricated using compression molding, which is suitable for these composites as it allows better handling of natural fibers and good distribution within the plastic matrix. The pressing cycle was conducted using a Manual Twist Heat Press Machine at a temperature of T = 180 °C and a pressure of P = 5 MPa. The pellets were pressed for 10 min under heat and for 90 min in cold to achieve total fusion of the composite material and obtain a completely flat panel.

The final size of the composite panel was 180 mm × 140 mm × 3 mm (thickness). The formulations of the composite materials are presented in Table 1.

### 2.2. Material Characterization

To determine the tensile strength of the developed materials, tests were conducted using a WDW100 UTM universal testing machine with a 5 kN load cell, according to the ASTM D638 standard [31], at a constant speed of 5 mm/min and an ambient temperature of ~20 °C. Three replicates were tested for each composite material treatment.

To understand the structure of the individual molecules and the composition of the molecular mixtures of the materials, Fourier Transform Infrared Spectroscopy (FTIR) was performed. The FTIR spectra of the studied compounds were recorded with a resolution of 400 to 4000 cm^−1^ using a SHIMADZU Model IRSpirit spectrometer. Each of the evaluated composite materials was analyzed in the form of particles of ~1 mm.

Thermal stability analyses of the samples were performed using an SDT-Q600 thermogravimetric analyzer. Thermogravimetric Analysis (TGA) and Differential Scanning Calorimetry (DSC) tests were conducted in a platinum crucible under an inert nitrogen atmosphere at a heating rate of 10 °C/min. The test temperature range was from 10 °C to 320 °C.

For the scanning electron microscopy (SEM) analysis, composite materials with different composition percentages (HDPEr + 10% BF and HDPEr + 20% BF), as well as unaltered banana fiber and recycled high-density polyethylene samples, were collected. The samples were mounted with copper tape to improve conductivity. SEM analysis was performed using a ZEISS EVO MA10 scanning electron microscope.

These tests were conducted to analyze the composite material when subjected to tensile stress, to demonstrate the interfacial adhesion between the matrix and the reinforcement, and to assess the thermal stability of the composite material.

## 3. Results

The results of the tests carried out on the developed composite materials are presented below, showing the average tensile strength measurements from three replicates of each sample.

### 3.1. Tensile Strength Measurements

Table 2 presents the tensile strength properties of virgin HDPE reinforced with 10% by weight banana fiber (the control sample) and recycled HDPE composites reinforced with 10% and 20% by weight banana fiber.

**Figure 1:**
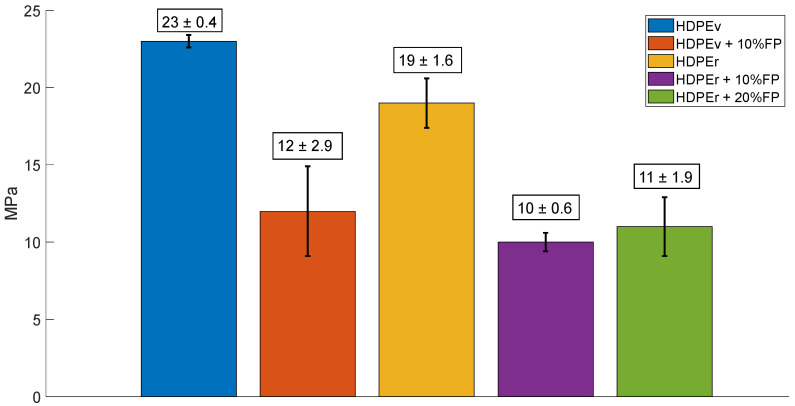
Tensile strength of the developed materials.

Figure 1 shows the tensile strength of the evaluated materials. Virgin high-density polyethylene (HDPEv) exhibited a tensile strength of 22.77 MPa and a strain of 16.9%. These results are attributed to the material not having been subjected to any previous heating processes. In comparison, recycled HDPE (HDPEr), which had been processed at least twice, showed a tensile strength of 18.7 MPa and a strain of 4.3%, demonstrating a significant decrease of 12.6% in strain.

The virgin HDPE composite reinforced with 10% banana fiber (HDPEv + 10% BF) had a tensile strength of 15.8 MPa, representing a decrease of 46.6% compared to virgin HDPE. For recycled HDPE reinforced with 10% and 20% banana fiber, similar tensile strengths were observed: HDPEr + 20% BF had a tensile strength of 11.88 MPa, and HDPEr + 10% BF had a tensile strength of 10.34 MPa. However, there was a notable difference in their strain percentages. Specifically, HDPEr + 20% BF exhibited a decrease of 44.7%, and HDPEr + 10% BF showed a decrease of 36.5% in tensile strength compared to recycled HDPE.

Bhupendra Kumar Singha and Ujendra Kumar Komal investigated high-density polyethylene matrix composites (both recycled and virgin) reinforced with 20% plantain fiber in their study “Development of Banana Fiber-Reinforced Composites from Plastic Waste” [32]. Their results indicated that the tensile strength of virgin HDPE reinforced with 20% banana fiber decreased by 19.32% compared to unreinforced virgin HDPE. Similarly, the tensile strength of recycled HDPE composites reinforced with 20% banana fiber decreased by 3.21% compared to unreinforced recycled HDPE. They concluded that this reduction in tensile strength is due to the incorporation of short natural fibers, which promote multiple nucleations.

Comparing the results obtained, it can be confirmed that the tensile strength of the evaluated materials is inversely proportional to the amount of short banana fiber present in the composite; that is, higher banana fiber content leads to lower tensile strength. Additionally, as the banana fiber content in high-density polyethylene increases, the percentage of strain also rises, as demonstrated in Table 2.

### 3.2. FTIR Analysis

FTIR is an effective method for detecting the functional groups present in raw materials and the developed composites (HDPEv + 10% BF, HDPEr + 10% BF, and HDPEr + 20% BF), aiding in the investigation of the characteristics of banana fiber and recycled HDPE. The characteristic infrared bands of the functional groups are listed in Table 3.

Analysis of the banana fiber spectra reveals distinct bands corresponding to the components of cellulose, hemicellulose, and lignin fractions characteristic of lignocellulosic fibers. The broad absorption band at 3362 cm^−1^ is attributed to the O–H stretching vibration of hydroxyl groups, indicating the presence of absorbed water, free phenols, and primary and secondary aliphatic alcohols found in cellulose, hemicellulose, and lignin [33,34]. The presence of this hydroxyl group stretching band, associated with cellulose, can contribute to fibers with higher strength [35].

The absorption bands at 2913 cm^−1^ and 2850 cm^−1^ correspond to the stretching vibrations of the aliphatic C–H group, specifically CH₂, indicating the presence of cellulose and hemicellulose. Previous studies have reported an absorption band at 1730 cm^−1^ corresponding to the stretching vibration of the carbonyl group C=O, attributed to acetyl groups typically present in hemicellulose. Additionally, absorption bands at 1595 cm^−1^ and 1315 cm^−1^ correspond to the angular deformation vibration of C=C associated with aromatic rings, the asymmetric deformation vibration of the C–H group, and the stretching vibration of C–O, respectively, due to the presence of lignin [36,37]. However, the spectrum of banana fiber in this study does not show these bands, which may be due to the alkaline treatment performed to remove hemicellulose and lignin.

The absorption band at 1035 cm^−1^ corresponds to the stretching vibration of O–H, directly associated with the cellulose in banana fiber [38,39,40].

Regarding the spectra containing HDPE, bands within 3000 cm^−1^ to 2800 cm^−1^ are linked to C–H stretching vibrations, corresponding to hydrocarbons present in HDPE [41]. The bands observed at 1472 cm^−1^ and 717 cm^−1^ are associated with the C–H bond representing phenyl ring substitution, which becomes evident in the presence of cellulose, indicating the incorporation of natural fiber. Additionally, a very low intensity is observed during the pyrolysis of polymers [42].

**Figure 2:**
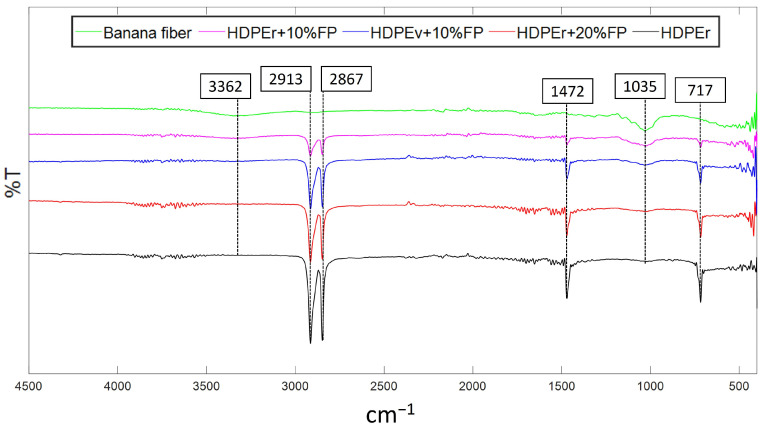
FTIR spectra of the composite materials.

The results of the FTIR analysis of the composite materials are shown in Figure 2.

### 3.3. Thermal Analysis

**Figure 3:**
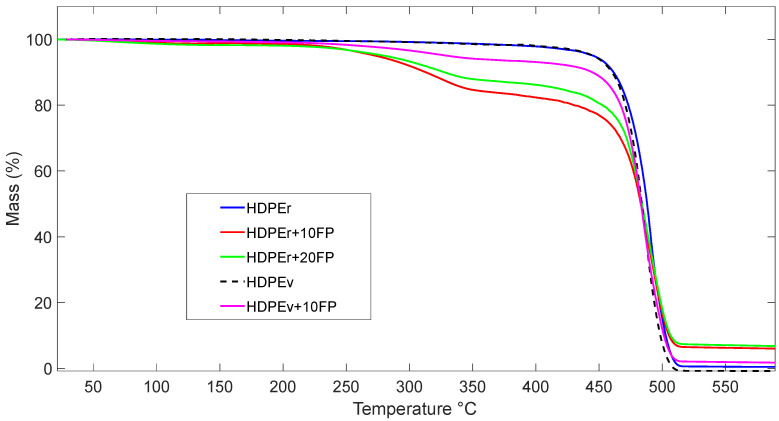
TGA curves of HDPE and its compounds.

Figure 3 presents the TGA curves of the five types of materials evaluated. The sample HDPEr + 10% BF exhibits high thermal stability below 200 °C, retaining 6% residual mass up to 600 °C, with a single mass loss peak starting around 350 °C. This peak corresponds to the degradation of banana fiber used as reinforcement in the polymer. High-density polyethylene materials reinforced with natural fibers typically display a double degradation curve: the first corresponds to the natural fiber and the second to the high-density polyethylene.

In Figure 3, two stages of degradation are observed in the three samples of fiber-reinforced composite materials (HDPEr + 10% BF, HDPEr + 20% BF, and HDPEv + 10% BF). The first stage occurs between 25 °C and 125 °C, with a mass variation of ~2.0%, corresponding to the loss of physically adsorbed water. In the temperature range of 125 °C to 300 °C, thermal degradation comprises multiple stages, including the degradation of pectin and hemicellulose, followed by cellulose and lignin. The second stage of degradation is observed between 300 °C and 550 °C, with a mass change of approximately 6.0%, which can be attributed to the loss of HDPE. The higher mass loss in the first stage suggests that the materials contain minimal physically adsorbed water, specifically in the natural fiber.

The results for all samples indicate that thermal degradation occurs through a single continuous process, demonstrating improved thermal properties.

Thermal degradation parameters, such as initial degradation temperature (Initial T) at 5% mass loss, maximum degradation temperature (T_max), final decomposition temperature (Final T), and residual mass at 600 °C, are listed in Table 4.

The thermogravimetric analysis indicates that the initial decomposition temperatures and final decomposition temperatures for virgin HDPE were 447.9 °C and 588.73 °C, respectively, while for recycled HDPE these temperatures were in the range of 445 °C to 588 °C, demonstrating a degree of similarity. The incorporation of banana fiber into recycled HDPE decreased the initial degradation temperature to between 270 °C and 280 °C, whereas for pure HDPE with 10% banana fiber, an initial degradation temperature of 330.4 °C was observed. However, the final decomposition temperature for all samples was found to be between 500 °C and 590 °C. Since banana fiber begins to degrade from 200 °C, the fiber is consumed first, followed by the polymer, which explains the decrease in the thermal stability of HDPE.

The change in the initial degradation temperature is possibly related to a decrease in the permeability and/or diffusivity of volatile decomposition products of the polymers due to the incorporation of inorganic particles. In general, the incorporation of banana fiber into HDPE was beneficial; although the banana fiber incinerates after 200 °C, the entire composite material maintains thermal resistance, exhibiting behavior similar to the polymeric matrix without reinforcement, which is favorable for its use. The residual mass of the recycled HDPE sample with 10% banana fiber at 600 °C is nearly identical to that of recycled HDPE with 20% banana fiber, with residual mass percentages of 6.00% and 6.76%, respectively.

Differential scanning calorimetry (DSC) testing was performed on five samples: HDPEr, HDPEr + 10FP, HDPEr + 20FP, HDPEv and HDPEv + 10FP. The test had a maximum temperature of 320 °C, with an endothermic event observed at approximately 150 °C.

**Figure 4:**
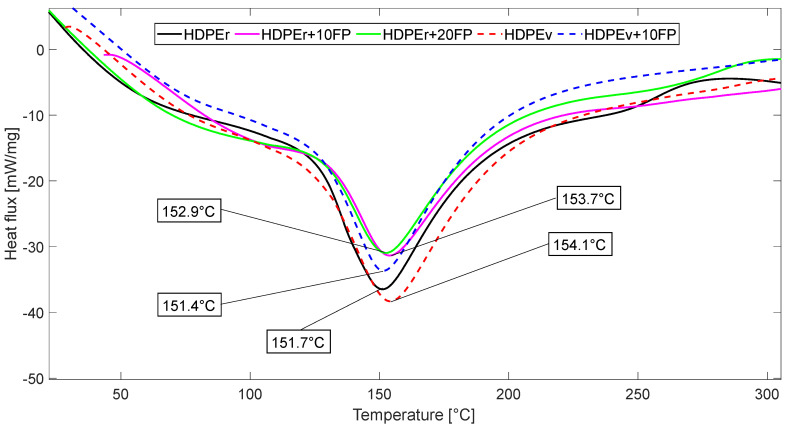
DSC thermogram of heating performed on the evaluated materials.

DSC analysis was conducted to investigate the thermal characteristics of the developed materials. Figure 4 shows the DSC curves of HDPEr, HDPEr + 10FP, HDPEr + 20FP, HDPEv and HDPEv + 10FP, indicating the obtained melting temperatures (Tm). The addition of banana fiber to recycled HDPE led to a slight increase in Tm values, suggesting that the crystallinity of the composite remained stable. This increase in crystallinity may be attributed to the nucleating effects of the banana fibers, which act as templates for the formation of crystalline regions in the HDPE matrix. The presence of polar groups on the surface of the banana fibers causes this nucleating effect, interacting with the HDPE matrix and promoting crystallization [43].

The DSC analysis also showed no significant change in the melting peak of the composites, with melting temperatures between 151 °C and 154 °C, indicating thermal stability of the composites. This thermal stability could be attributed to the interfacial bonding between the fiber reinforcement and the HDPE matrix, which reduces the mobility of the polymer chains without altering the melting temperature. The DSC analysis demonstrated that the incorporation of banana fibers into recycled HDPE did not significantly alter the thermal properties of the composites.

The thermal analysis of virgin HDPE involved gradually heating the material from 10 °C to 320 °C at a constant rate of 20 °C/min. Since the composites were produced from a thermoplastic matrix, the DSC analysis was carried out up to 320 °C, and the expected results were obtained before reaching this temperature. The resulting graph showed a negative peak, indicating that HDPE absorbed energy during the heating process. This observation suggests the occurrence of an endothermic event within the HDPE material. The maximum melting temperature was found to be 157.1 °C, at which point the material transitioned from being relatively hard to rubbery. This is because HDPE has a melting point within the range of approximately 120 °C to 180 °C, with an average melting point around 140 °C. At this temperature, the HDPE material softens and becomes rubbery, facilitating its conversion into a composite material. The sharp and intense peak around 150 °C indicates that a large amount of energy is absorbed, corresponding to the presence of –C–C– bonds in the composite. The peak at 154.1 °C corresponds to the maximum energy absorption of virgin HDPE, with an enthalpy of 290 J/g [44]. Similarly, banana fiber-reinforced composites subjected to thermal analysis showed a negative peak between 151 °C and 153 °C, indicating an endothermic event as the material absorbs energy. The energy absorbed by the banana fiber-reinforced composites was found to be around 82.36 J/g.

Differential Scanning Calorimetry is a highly sensitive technique for detecting enthalpy changes, including crystallization. During DSC experiments, the heating process induces oriented polymer chain structures to form larger crystals before the melting point.

At the maximum melting temperature (Tm), the composite transitions from a relatively hard material to a rubberier state. The energy required for this phase change, known as the heat of fusion, is estimated to be 149.6 J/g for the developed materials. As the temperature increases beyond the maximum melting temperature of 254.9 °C, the composite exhibits increased flexibility, allowing the amorphous structure to reorganize into a lower-energy crystalline form. However, when the temperature is rapidly increased further, the composite undergoes a complete transition to a fully amorphous polymer state.

### 3.4. Scanning Electron Microscopy (SEM)

**Figure 5:**
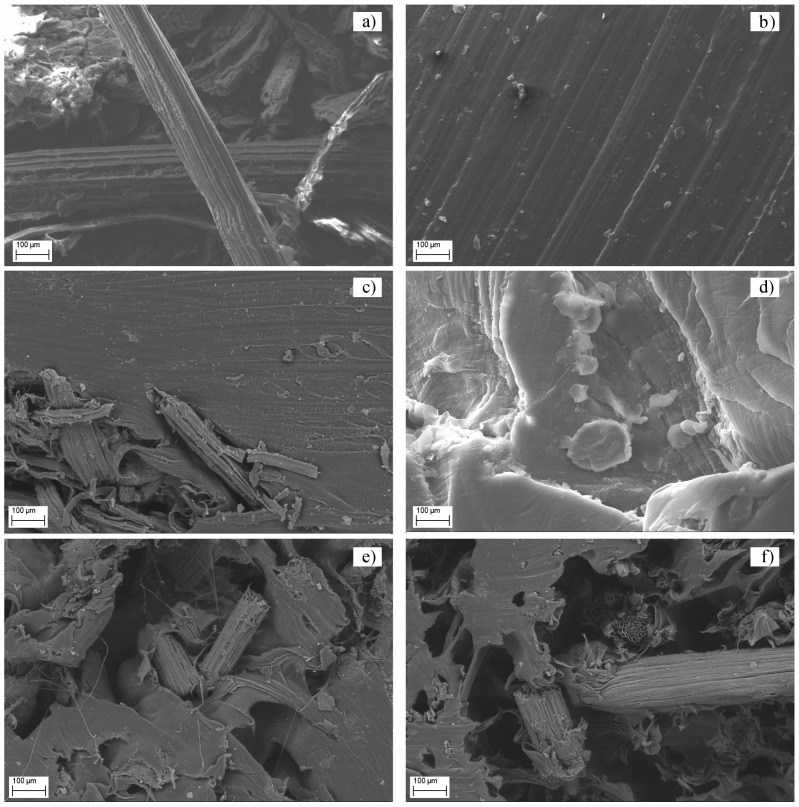
SEM micrographs of HDPE composite samples: (**a**) banana fiber, (**b**) virgin high-density polyethylene, (**c**) virgin high-density polyethylene with 10% banana fiber, (**d**) recycled high-density polyethylene, (**e**) recycled high-density polyethylene with 10% banana fiber, and (**f**) recycled high-density polyethylene with 20% banana fiber.

Scanning electron microscopy (SEM) was performed to determine the microstructural causes leading to the fracture of the material. After tensile testing, SEM analysis of the fractured surfaces of the composite materials was conducted to observe the interfacial adhesion between the matrix and the banana fibers, as shown in Figure 5.

Figure 5a,b,d show SEM images of the raw materials used to prepare the composites. In contrast, Figure 5c,e,f depicts SEM images where the short banana fibers are aggregated and unevenly dispersed within the polymeric matrix. This indicates poor adhesion between the polymeric phase and the reinforcement phase in the composites HDPEv + 10% BF, HDPEr + 10% BF, and HDPEr + 20% BF.

Figure 5c shows that, despite the agglomeration and inhomogeneous distribution of banana fibers within the polymeric matrix, no cavities are formed, suggesting that the fibers have not separated from the matrix. The absence of separation indicates good interfacial bonding between the fibers and the polymer. However, the presence of fiber agglomerates could act as stress concentrators, potentially contributing to fracture under tensile load.

Figure 5e,f illustrates the microstructure of recycled HDPE composites reinforced with 10% and 20% short banana fiber, respectively. The micrographs reveal that the failure modes under tensile load include fiber pullout and breakage, indicating moderate interfacial bonding between the fibers and the polymer.

Furthermore, Figure 5 reveals that the banana fibers are not evenly distributed within the HDPE matrix. As a result, the fibers could not effectively enhance the performance of the composite material, particularly in terms of tensile strength, as observed in this study.

These findings are consistent with those reported by Praveen Nagarajan Durai and Kathir Viswalingam in their paper “Study of the Mechanical Properties of HDPE Composites Reinforced with Banana Peduncle Fiber” [24]. Their results evidence that the morphology of HDPE compounds reinforced with short banana fiber exhibited improved adhesion in treated samples, where the rough surface of the banana stem fiber was achieved through alkaline treatment. In contrast, untreated samples displayed voids and fiber breakdown due to poor adhesion between the fiber and the matrix.

In the study by Praveen Nagarajan, it was determined that alkaline treatment increased the roughness of the fiber surface, leading to better bonding between the fibers and HDPE, as observed in SEM micrographs of the fracture surface. This indicates that efficient adhesion at the fiber–matrix interface is a key factor in improving impact resistance.

Considering the data obtained in the present study, it was observed that alkaline treatment of short banana fiber enhances adhesion with the polymeric matrix, as seen in Figure 5a. Therefore, this chemical treatment can be considered the most suitable for banana fiber reinforcement. Additionally, impurities were observed in the recycled high-density polyethylene, even after washing with NaOH.

## 4. Discussion

In this study, a comprehensive evaluation of the tensile strength of various polymeric materials reinforced with short banana fibers was conducted. The aim was to observe the behavior of the physical and morphological properties of the developed materials and to assess the influence of fiber incorporation on the resulting composites. The results indicate a gradual reduction in tensile strength with increasing amounts of banana fiber in the composite materials. This reduction is attributed to the tendency of the fibers to agglomerate within the polymeric matrix, despite a seemingly homogeneous mixture, leading to stress concentration points. While this trend is observed across all materials, certain composites exhibit a more pronounced response, indicating varying sensitivities to different concentrations of banana fiber loading [45,46].

Notably, the observation of higher reinforcement concentrations within the polymer matrix reveals an inversely proportional relationship between tensile strength and banana fiber loading [32,47]. This phenomenon is attributed to poor adhesion between the natural fibers and the HDPE matrix, which can generate weak points where stresses concentrate, leading to premature failures. Furthermore, high fiber loading can result in a non-uniform distribution, creating stress concentration zones and acting as crack initiators [48]. The intrinsic properties of banana fibers, which may be less resistant than HDPE, also contribute to the reduction in overall composite strength. Additionally, the recycling process can degrade the mechanical properties of HDPE and hinder homogeneous mixing with the fibers [10]. Finally, a high proportion of fibers can decrease the volume of matrix available to properly encapsulate them, worsening dispersion and increasing susceptibility to failure under load. Consequently, an increase in banana fiber loading correlates with a decrease in the tensile strength of the composite [46,47].

The findings of this study not only enrich our understanding of the influence of short banana fiber reinforcement loading on polymeric materials but also provide valuable insights into their mechanical, physical, and morphological properties and behavior. These results support the notion that the tensile strength of composites is directly correlated with the amount and nature of the incorporated fibers. This is consistent with the study by Bhupendra Kumar Singh and Ujendra Kumar Komal, which indicated that the addition of banana fibers significantly reduced the tensile strength of both virgin and recycled HDPE [25].

## 5. Conclusions

Thermoplastic composite materials were developed from agro-industrial waste by reinforcing recycled high-density polyethylene (HDPE) waste with banana fiber waste. Mechanical evaluation demonstrated that the tensile strength and modulus of elasticity depend on the content of short banana fibers and the type of HDPE used. Furthermore, it was shown that higher fiber loading leads to a lower modulus of elasticity, as evidenced by the higher elastic modulus of the unreinforced polymeric matrix compared to the composites with banana fiber fillers. Thermogravimetric analysis revealed faster decomposition of the composites compared to pure polymers (virgin and recycled HDPE), possibly due to the addition of banana fibers. Thermal analysis also established a temperature limit of 240 °C for processing the compounds without thermal degradation. HDPE recycling did not significantly affect the thermal behavior of the manufactured composite materials compared to pure HDPE. However, recycling significantly affected the mechanical properties of the prepared composites, decreasing their tensile strength. Considering all these factors, the described method holds great promise for replacing expensive polymer-based composite panels, aiming to achieve more competitive and environmentally sustainable materials.

## Figures and Tables

**Table 1:** Formulations of high-density polyethylene reinforced with banana fiber (percentages by weight).

Composite Samples	HDPE (%)	Banana Fiber (%)
HDPEr + 10% FP	90	10
HDPEr + 20% FP	80	20
HDPEv + 10% FP	90	10

Note: The acronyms FP, HDPEv and HDPEr refer to banana fiber, virgin high-density polyethylene, and recycled high-density polyethylene, respectively.

**Table 2:** Tensile strength properties of banana fiber-reinforced HDPE composites.

Composite Samples	Tensile Strength (MPa)	Strain %	Elastic Module (GPa)
HDPEv	23 ± 0.4	5.8	1.3 ± 0.2
HDPEv + 10% FP	15 ± 2.9	15.9	0.3 ± 0.01
HDPEr	19 ± 1.6	4.1	1.2 ± 0.08
HDPEr + 10% FP	10 ± 0.6	5.6	0.3 ± 0.06
HDPEr + 20% FP	11 ± 1.9	7.9	0.3 ± 0.04

Note: BF = Banana Fiber; HDPEv = Virgin High-Density Polyethylene; HDPEr = Recycled High-Density Polyethylene.

**Table 3:** Characteristic functional groups of composite materials and their corresponding frequencies.

Infrared Band (cm^−1^)	Vibration	Corresponding Compounds
3500~3100	O–H stretching	Water, free phenols, primary and secondary aliphatic alcohols
3000~2800	C–H stretching	Hydrocarbons
1300~1000	C–O stretching	Ethers, alcohols, phenols
800~600	C–H stretching	Hydrocarbons

**Table 4:** Thermal degradation parameters.

Sample	Initial T (°C)	Max T (°C)	Final T (°C)	Residual Mass %
HDPEr	445.43	495.35	588.73	0.43
HDPEr + 10FP	273.48	495.37	588.87	6.00
HDPEr + 20FP	280.52	505.64	589.14	6.76
HDPEv	447.90	492.09	508.20	0.00
HDPEv + 10FP	330.40	491.36	589.00	1.73

## Data Availability

The original contributions presented in the study are included in the article, further inquiries can be directed to the corresponding author.
